# Reviewed article: Silva LAN, Delpino FM, Figueiredo LM, Thumé E, Tomasi E, Facchini LA, Nunes BP. Adult use of health services: a cross-sectional population-based study, Pelotas, Brazil, 2012 and 2021. Epidemiol Serv Saude. 2026:35;e20250460

**DOI:** 10.1590/S2237-96222026v35e20250460.a

**Published:** 2026-04-20

**Authors:** Johnnatas Mikael Lopes

**Affiliations:** 1 Universidade Federal do Vale do São Francisco, Paulo Afonso, BA, Brazil Universidade Federal do Vale do São Francisco Paulo Afonso BA Brazil

## Peer review A

## Questionnaire

Does the manuscript contain new and significant information to justify publication? Yes

Does the Abstract (Summary) clearly and accurately describe the content of the article? No

Is the problem significant and concisely stated? Yes

Are the methods described comprehensively? Yes

Are the interpretations and conclusions justified by the results? Yes

Is adequate reference made to other work in the field? Yes

## Manuscript Structure: 

Length of article is: Adequate

Number of tables is: Too little

Number of figures is: Adequate

Please state any conflict(s) of interest that you have in relation to the review of this paper: None

Interest: Good

Quality: Average

Originality: Below Average

Overall: Average

Recommendation: Minor Revision

## Comments

O manuscrito apresentado nessa submissão aborda tema interessante para a gestão sanitária e a elaboração de políticas públicas sobre uso de serviços de saúde. Existem alguns cometários metodológicos que precisam ser ressaltados e, possivelmente abordados: 1. Entendo que o desenho com confronto de duas pesquisas transversais se aproxima mais de um estudo em painel do que relatar como sendo apenas desenho seccional. 2. A comparação de medidas absolutas, como o indicador de desigualdade entre as duas coletas, levanta um problema caracteristico de estudos com ponderações amostrais diferentes. Como pacotes estatísticos para análise de amostras complexas permitem a inserção de apenas uma variável de peso amostral. Hove uma harmonização do peso amostral das duas coletas? Se sim, especifique no texto metodológico como foi realizado. Se não, explique as limitiações que isso implica na capacidade inferencial dos achados. Após esses esclarecimentos será possível analisar a discussão dos achados. 

